# Dose-response effects of corn-derived deoxynivalenol on nursery pig feed intake and growth performance

**DOI:** 10.1093/tas/txag104

**Published:** 2026-07-11

**Authors:** Thomas A Crome, Darlene J Bloxham, Scott L Radke, Fredrik B Sandberg, Nicholas K Gabler

**Affiliations:** Department of Animal Science, Iowa State University, Ames, IA 50011, United States; Adisseo USA, Inc, Alpharetta, GA 30022, United States; Department of Veterinary Diagnostic and Production Animal Medicine, Iowa State University, Ames, IA 50011, United States; Furst McNess Company, Rockford, IL 61108, United States; Department of Animal Science, Iowa State University, Ames, IA 50011, United States

**Keywords:** deoxynivalenol, feed intake, nursery pig, performance, titration

## Abstract

Deoxynivalenol (DON) is a trichothecene mycotoxin commonly contaminating swine diets and is known to impair nursery pig performance through anorectic effects. The objective of this study was to characterize the dose-response effects of increasing dietary DON concentrations (0.2 to 5.1 mg/kg) that were naturally added through increasing levels of DON-contaminated corn on growth performance in modern nursery pigs using a seven-point titration design. A total of 112 pigs (9.1 ± 0.37 kg BW) were assigned to one of seven dietary treatments (*n* = 8 pens/treatment; 2 pigs/pen) and fed experimental diets for 24 d (Phase 1: d 0–14; Phase 2: d 14–24). Growth performance declined linearly during both Phase 1 and Phase 2 (*P* < 0.001). Across the 24-d period, increasing DON reduced overall BW, ADG, and ADFI in a linear manner (*P* < 0.001), with no quadratic responses detected (*P* > 0.100) DON concentration explained 39%, 48%, and 58% of the variation in final BW, ADG, and ADFI, respectively. Each 1 mg/kg increase in DON was associated with a 1.13 kg reduction in final BW, a 0.04 kg/d reduction in ADG, and a 0.07 kg/d reduction in ADFI. Overall Gain:Feed was not linearly related to DON concentration (*P* = 0.214). Two-segmented and linear-plateau regression analysis provided minimal improvement over linear models, indicating limited to no evidence for a biological breakpoint within the evaluated range. These findings demonstrate a clear, linear, dose-dependent suppression of voluntary feed intake and growth performance in nursery pigs exposed to 0.2 to 5.1 mg/kg DON, without evidence of a discrete intake threshold.

## Introduction

Deoxynivalenol (DON) is a type B trichothecene mycotoxin produced primarily by *Fusarium graminearum* that commonly infects cereal grains in temperate growing regions (Radke et al. 2025). In North America, DON is one of the most prevalent mycotoxins contaminating corn and wheat-based swine diets, with concentrations varying substantially across years and geographic regions (Streit et al. 2013). A recent survey of U.S. new crop corn reported that DON was detected in 76% of grain samples (Weaver et al. 2021). Swine are considered the most sensitive domestic livestock species to DON, particularly during the nursery period (Andretta et al. 2012), when pigs exhibit heightened susceptibility to feed intake suppression and growth impairment (Eriksen and Pettersson 2004; Pestka 2010). Corn is typically an ingredient that makes up a large percentage of many U.S. pig diets, and even moderate DON contamination can impair performance. Additionally, DON is further concentrated in dried distillers’ grains with solubles from contaminated corn (Frobose et al. 2015a). The primary biological consequence of dietary DON exposure in pigs is a reduction in voluntary feed intake, which subsequently depresses average daily gain and final body weight (Friend et al. 1986; House et al. 2002). The United States Food and Drug Administration currently does not have advisory levels for DON in corn itself, only aflatoxin (USDA 2023). However, the most current guidance document states that 5 mg/kg DON on grains and grain by-products destined for swine with the added recommendation that these ingredients not exceed 20% of their diet, which equates to ∼1 mg/kg DON in finished feed (FDA 2010). In Europe, the guidance value for DON in swine feed is 0.9 mg/kg set by the European Food Safety Authority (EFSA COMTAM 2017).

Studies have demonstrated that dietary DON concentrations exceeding 1 mg/kg can negatively impact pig growth performance (Eriksen and Pettersson 2004), with more pronounced suppression observed as DON concentration increases (Rotter et al. 1996; Frobose et al. 2015a; Wilson et al. 2022). However, most performance-based investigations in swine have relied on single- or two-dose comparisons, typically contrasting a control diet with a diet containing a single elevated DON concentration (Chaytor et al. 2011; Alizadeh et al. 2015; Frobose et al. 2015b). While these studies confirm that DON impairs pig performance, they provide limited definition regarding the quantitative shape of the dose-response relationship across practical dietary concentrations. Wellington et al. (2020) modeled the relationship of average daily gain to DON exposure (0.1, 1.3, 3.6, and 5.7 mg/kg, respectively) in growing pigs, reporting decreased weight gain as DON concentration increased. This agrees with Andretta et al. (2012), who reported that for each mg/kg intake of DON, a 0.28% reduction in weight gain can be seen in growing pigs.

Establishing the quantitative relationship between dietary DON concentrations and nursery pig growth performance is essential to define biologically meaningful exposure thresholds and to provide a foundation for subsequent mechanistic and mitigation investigations. Therefore, the objective of this study was to characterize the dose-response effects of increasing dietary DON concentrations (0.2 to 5.1 mg/kg), which were naturally added through increasing levels of contaminated corn, on feed intake and growth performance in nursery pigs. We hypothesized that increasing dietary DON would reduce nursery pig feed intake and growth in a nonlinear manner, consistent with a biological breakpoint or threshold response.

## Materials and methods

Research was conducted at the Iowa State University Swine Nutrition Research Farms (Ames, IA) from May to June 2025. All animal procedures were approved by the Iowa State University Institutional Animal Care and Use Committee (IACUC protocol #24–069) and were conducted in accordance with the Guide for the Care and Use of Agricultural Animals in Research and Teaching (FASS 2010).

### Animals, housing, experimental design and diets

A total of 112 mixed-sex pigs (9.1 ± 0.37 kg; Terminal Line 337 × Maternal Line 1050, PIC Inc., Hendersonville, TN), 10 d post-weaning, and sourced from a commercial sow farm, were utilized and housed at the Iowa State University Swine Nutrition Farm (Ames, IA). Pigs were allotted using a completely randomized design and placed into 56 pens containing one barrow and one gilt per pen. All pigs were individually tagged and weighed. Once placed, pens were randomly assigned to 1 of 7 dietary treatments (*n* = 8 pens/treatment) containing dose titrated concentrations of DON. Pigs were housed in partially concrete slatted pens (0.97 m × 1.83 m) equipped with one nipple drinker and a two-hole feeder. At all times, the pigs had ad libitum access to feed and water. Ventilation rates and temperature set points were evaluated and adjusted weekly by farm personnel to ensure consistent environmental control. Individual pig body weights (BW) and pen feeder weights were taken on day 0, 14, and 24 for the calculation of pen average daily gain (ADG), average daily feed intake (ADFI), and feed efficiency (Gain:Feed; G:F) for phase 1 (14 d), phase 2 (10 d), and overall (d 0–24) of the study.

Experimental dietary treatments were formulated and manufactured as standard corn-soybean meal mash diets equal in energy and SID amino acids (Table 1). High DON corn (20 mg/kg) sourced from Ohio was used at the expense of clean corn (0.20 mg/kg) in the formulation to achieve target DON concentrations ranging from 0.30 to 5.25 mg/kg using a seven-point dose titration methodology. Complete feed DON concentrations (mg/kg) were formulated at 0.75 mg/kg increments to targets of < 0.30, 0.75, 1.50, 2.25, 3.00, 4.50 and 5.25 mg/kg (Table 1). Each diet was sampled at mixing and submitted to the Iowa State Veterinary Diagnostic Laboratory (Ames, IA, USA) for complete mycotoxin profiling and DON verification using LC-MS/MS standard testing procedures before the beginning of the study. All diets were within 0.5 mg/kg of their formulated DON concentration (Table 2). Aflatoxin B1, aflatoxin B2, aflatoxin G1, aflatoxin G2, fumonisin B1, fumonisin B2, ochratoxin A, T-2 toxin, HT-2 toxin, and alpha-zearalenol were not detectable or below detection limits. Diets were formulated based on NRC (2012) amino acid and energy recommendations for the size of pig used in this growth assay and were nutritionally equivalent across treatments, ensuring that observed performance responses could be attributed primarily to differences in dietary DON concentration rather than changes in nutrient composition.

**Table 1:** Ingredient and nutrient composition of experimental diets used to evaluate the dose titration effects of corn-derived deoxynivalenol (DON) on nursery pig growth performance, as-fed basis.

	DON mg/kg						
**Ingredient, %**	0.30	0.75	1.50	2.25	3.00	4.50	5.25
**Corn**	63.13	60.13	56.12	52.11	48.10	40.08	36.07
**High DON corn^a^**	–	3.00	7.01	11.02	15.03	23.05	27.06
**Soybean meal, 45% CP**	31.70	31.70	31.70	31.70	31.70	31.70	31.70
**Soybean oil**	1.00	1.00	1.00	1.00	1.00	1.00	1.00
**Limestone**	0.89	0.89	0.89	0.89	0.89	0.89	0.89
**Monocalcium phosphate 21%**	1.09	1.09	1.09	1.09	1.09	1.09	1.09
**Salt**	0.50	0.50	0.50	0.50	0.50	0.50	0.50
**L-lysine HCl**	0.57	0.57	0.57	0.57	0.57	0.57	0.57
**DL-methionine**	0.23	0.23	0.23	0.23	0.23	0.23	0.23
**L-threonine**	0.16	0.16	0.16	0.16	0.16	0.16	0.16
**L-tryptophan**	0.05	0.05	0.05	0.05	0.05	0.05	0.05
**L-valine**	0.13	0.13	0.13	0.13	0.13	0.13	0.13
**L-isoleucine**	0.09	0.09	0.09	0.09	0.09	0.09	0.09
**Vitamin premix^b^**	0.23	0.23	0.23	0.23	0.23	0.23	0.23
**Trace mineral premix^c^**	0.15	0.15	0.15	0.15	0.15	0.15	0.15
**Zinc oxide, 72% Zn**	0.05	0.05	0.05	0.05	0.05	0.05	0.05
**Phytase^d^**	0.03	0.03	0.03	0.03	0.03	0.03	0.03
***Calculated Composition***							
** ME^e^, Mcal/kg**	3.37	3.37	3.37	3.37	3.37	3.37	3.37
** Crude protein, %**	20.0	20.0	20.00	20.0	20.0	20.0	20.0
** Total calcium, %**	0.65	0.65	0.65	0.65	0.65	0.65	0.65
** Available phosphorus, %**	0.45	0.45	0.45	0.45	0.45	0.45	0.45
** Calcium:available phosphorus**	1.44	1.44	1.44	1.44	1.44	1.44	1.44
** SID^f^ Lysine, %**	1.33	1.33	1.33	1.33	1.33	1.33	1.33
** g SID Lysine:ME**	3.95	3.95	3.95	3.95	3.95	3.95	3.95

a Corn containing 20 mg/kg deoxynivalenol.

b Vitamin premix provided 6125 IU vitamin A, 700 IU vitamin D3, 50 IU vitamin E, 30 mg vitamin K, 0.05 mg vitamin B12, 11 mg riboflavin, 56 mg niacin, and 27 mg pantothenic acid per kilogram of diet.

c Trace mineral premix provided 9 mg Cu (as CuSO_4_), 120 mg Fe (as FeSO_4_), 0.21 mg I (as Ca(IO_3_)_2_), 6.75 mg Mn (as MnSO_4_), 120 mg Zn (as ZnSO_4_), and 0.23 mg Se (as Na_2_SeO_3_) per kilogram of diet.

d Optiphos 2500; Huvepharma, Peachtree City, GA, USA. Minimum activity of 750 phytase units (FTU)/kg complete feed providing 0.15% available phosphorus release.

e ME, Metabolizable energy.

f SID, Standardized ileal digestibility.

**Table 2:** Analyzed mycotoxin concentration of experimental diets, as fed basis.

	DON mg/kg^a^						
	0.30	0.75	1.50	2.25	3.00	4.50	5.25
**Deoxynivalenol, mg/kg**	0.2	0.9	1.7	2.4	3.1	4.0	5.1
**3-Acetyl Deoxynivalenol, µg/kg**	−^b^	15.3	25.5	34.6	49.5	59.9	81.4
**Zearalenone, mg/kg**	–	–	–	–	0.5	0.2	0.4

a Formulated deoxynivalenol (DON) mg/kg level using diets spiked with high DON-sourced corn at the expense of clean corn.

b -, not detected or below limit of detection along with aflatoxin B1, aflatoxin B2, aflatoxin G1, aflatoxin G2, fumonisin B1, fumonisin B2, ochratoxin A, T-2 toxin, HT-2 toxin, and alpha-zearalenol.

### Statistical analysis

Data were analyzed in R (v4.3.3; (R Core Team 2024)) using linear models (lm function) with dietary treatment as a fixed effect for growth performance parameters including BW, ADG, ADFI, and G:F. Pen was the experimental unit for all analyses. Model assumptions were evaluated at the pen level using studentized residuals, and pens with an absolute studentized residual greater than 3 were considered outliers and removed on a per-variable basis prior to estimation of treatment means. Treatment means were evaluated using orthogonal polynomial contrasts to assess linear and quadratic trends across increasing dietary DON concentrations using the contrast function. To evaluate potential biological thresholds, linear, two-segment, and linear-plateau regression models were compared using Akaike’s Information Criterion (AIC) and Bayesian Information Criterion (BIC) to assess improvements in model fit for ADG. The 1.7 mg/kg treatment level did not follow the overall monotonic dose-response pattern and exerted disproportionate influence on regression model fit, being well outside 95% confidence interval (CI) range. Regression analyses were conducted both with and without this treatment level, with primary interpretation based on models excluding the 1.7 mg/kg treatment. Regression equations were derived for overall growth performance parameters. Treatment differences were considered significant at *P* ≤ 0.05 and a tendency at 0.05 < *P* ≤ 0.10.

## Results

The complete randomization of pigs across treatments resulted in initial BW not differing among DON treatments (*p* > 0.100; data not shown). The phase 1 and phase 2 growth performance data are presented in Table 3. Phase 1 ADG and ADFI decreased with increasing DON concentration by up to 52% and 44%, respectively (Linear *P* < 0.001). As such, G:F during phase 1 decreased by up to 13% as DON concentration increased (Linear *P* = 0.037). During Phase 2 (d 14–24), ADFI decreased by up to 39% as DON increased (Linear *P* < 0.001). Growth continued to be decreased by DON in a linear (*P* < 0.001) and quadratic (*P* = 0.034) manner by up to 22% decline across the titration treatments. Despite continued reduced feed intakes, G:F during Phase 2 increased with increasing DON concentration by up to 23% (Linear *P* < 0.001).

**Table 3:** Effect of corn-derived deoxynivalenol (DON) on nursery pig phase 1 and phase 2 growth performance and feed intakes.

	DON mg/kg^f^								*P*-value^g^	
	0.2	0.9	1.7	2.4	3.1	4.0	5.1	SEM	Linear	Quadratic
***Phase 1, d 0–14***										
** ADG, kg**	0.509^a^	0.469^ab^	0.259^de^	0.404^bc^	0.354 ^cd^	0.284^de^	0.245^e^	0.025	<0.001	0.150
** ADFI, kg**	0.638^a^	0.572^ab^	0.413 ^cd^	0.489^bc^	0.471^bc^	0.364^d^	0.355^d^	0.250	<0.001	0.103
** Gain:Feed**	0.795^ab^	0.818^a^	0.652^c^	0.829^a^	0.748^abc^	0.774^ab^	0.688^bc^	0.028	0.037	0.688
***Phase 2, d 14–24***										
** ADG, kg**	0.697^a^	0.714^a^	0.664^ab^	0.736^a^	0.644^ab^	0.535^b^	0.547^b^	0.032	<0.001	0.034
** ADFI, kg**	1.127^a^	1.048^ab^	0.927^bc^	1.047^ab^	0.888^bcd^	0.747 ^cd^	0.688^d^	0.046	<0.001	0.196
** Gain:Feed**	0.619^b^	0.684^ab^	0.715^a^	0.704^a^	0.674^ab^	0.716^a^	0.760^a^	0.021	<0.001	0.619

a,b,c,d,e Within row, treatment means without a common superscript differ as significant, *P* < 0.05.

f Analyzed DON mg/kg concentration in complete dietary treatments.

g Data were analyzed using linear and quadratic contrasts assessing trends across dietary treatments, *n* = 8 pens per treatment with 2 pigs per pen (1 barrow, 1 gilt).

**Figure 1:**
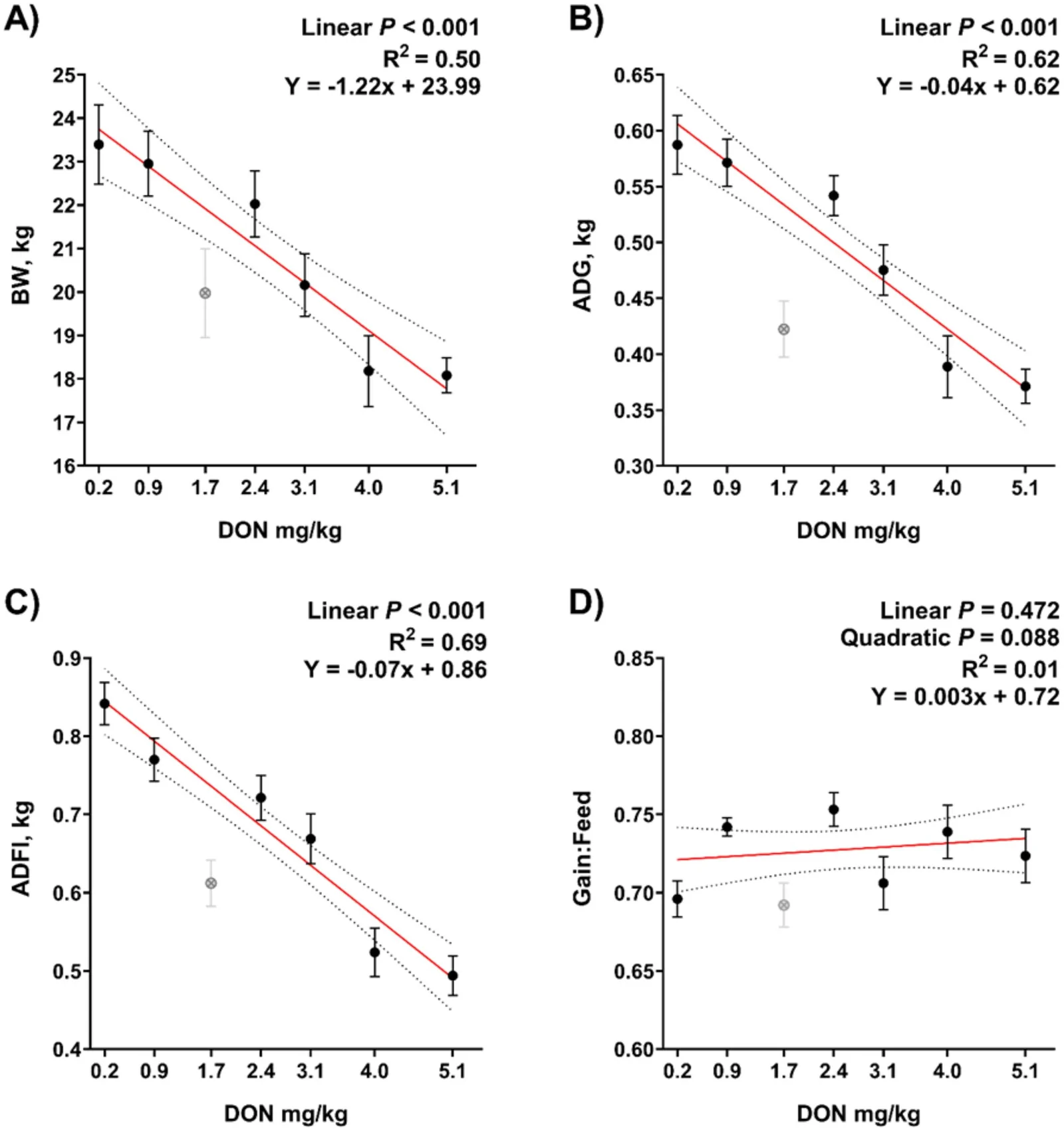
Dose-response effects of corn derived dietary deoxynivalenol (DON) on nursery pig growth performance. Panels depict DON’s impact on A) end body weight (BW) at d 24, and overall (d 0–24) average daily gain (ADG; B), C) average daily feed intake (ADFI), and D) Gain:Feed. No quadratic responses were observed for BW, ADG, or ADFI, *P* > 0.10. The 1.7 mg/kg treatment mean is displayed for transparency but was excluded from regression model fitting following pen-level outlier diagnostics.

A small number of pens were removed for Gain:Feed following prespecified outlier diagnostics outlined above: 1 pen from 5.1 mg/kg treatment and 2 pens from 3.1 mg/kg. The 1.7 mg/kg treatment mean did not follow the otherwise consistent dose-response pattern and influenced regression model fit; therefore, regression analyses were conducted with and without this treatment level, with models excluding it used for primary interpretation. A second mycotoxin screen was conducted on the 1.7 mg/kg treatment, and the results were the same, confirming accuracy of the analysis. Treatment means for all seven dietary DON concentrations are presented for transparency (Fig. 1). Over the entire 24 d study period, compared to the 0.2 mg/kg diet, increasing dietary DON concentrations reduced BW by 1.22 kg for each 1 mg/kg increase in dietary DON (Linear *P* < 0.001; *R*^2^ = 0.50; Fig. 1A). Similarly, overall ADG declined linearly with increasing DON (Linear *P* < 0.001; Fig. 1B), corresponding to a reduction of approximately 0.04 kg/d per 1 mg/kg DON increase (*R*^2^ = 0.62). Average daily feed intake exhibited the strongest correlation with DON concentration (*R*^2^ = 0.69), decreasing by 0.07 kg/d per mg/kg DON increase (Linear *P* < 0.001; Fig. 1C). Overall Gain:Feed was not linearly related to DON concentration (*P* = 0.472; Fig. 1D), however a quadratic tendency was observed (*P* = 0.088; Fig. 1D), and regression analysis demonstrated minimal correlation (*R*^2^ = 0.01).

One objective of this study was to determine whether a biological breakpoint existed within the tested DON range using segmented regression models. Linear, two-segment, and linear-plateau regression models were used to compare model fit. Model comparison indicated that linear models provided the best overall fit based on AIC and BIC for the effect of DON (mg/d) intake on ADG). Gain:Feed was not associated with dietary DON concentration (*R*^2^ = 0.01); therefore, breakpoint model comparisons were not conducted for this parameter. Due to the strong linear relationship of this dataset, no clearly identifiable break point was detected.

## Discussion

Deoxynivalenol is a prevalent Type B-trichothecene produced by *Fusarium* fungi that commonly contaminates cereal grains used in swine diets. Swine are recognized as one of the most sensitive livestock species to DON exposure, with reduced voluntary feed intake and subsequent growth depression consistently identified as the primary clinical manifestations at practical dietary concentration (Pestka 2010). The study objective was to characterize the quantitative dose-response relationship between increasing dietary DON concentrations (0.2 to 5.1 mg/kg) on nursery pig growth performance, with particular emphasis on determining whether responses follow a linear or non-linear threshold-based pattern within a commercially relevant exposure range. Unlike multiple previous studies that evaluated only one or two DON concentrations (Frobose et al. 2015a; Wilson et al. 2022), the seven-point titration design used here allowed for more precise characterization of the dose-response relationship across commercially relevant exposure levels with DON sourced from naturally contaminated corn. Current DON guidance values for swine diets are approximately 0.9 mg/kg in complete feed in Europe, whereas in the United States, FDA guidance limits contaminated grains to 5 mg/kg DON at a maximum of 20% of the diet, corresponding to approximately 1 mg/kg in finished feed (FDA 2010; EFSA COMTAM 2017). However, the FDA does not report the literature that is used to determine their guidance level whereas the EFSA summarized all of the data that went into their decision. Surveys of cereal grains and finished feeds frequently report DON contamination within or above this 0.9–5.0 mg/kg DON range, suggesting that nursery pigs may routinely encounter concentrations capable of impairing performance under commercial production conditions (Streit et al. 2013).

To our knowledge, few published studies have applied segmented regression to identify potential breakpoints where dietary DON concentration alters nursery pig performance (Dersjant-Li et al. 2003). Linear models provided the best overall fit for the relationship between DON mg/d intake and ADG. The absence of identifiable breakpoints in the segmented regression analysis and lack of significant quadratic responses further support the interpretation that performance responses to DON are proportional across the evaluated concentration range (0.2–5.1 mg/kg). Young et al. (1983) similarly reported linear reductions in ADFI and ADG in 7.1 kg pigs fed diets containing 0.14, 1.34, 2.55, and 5.12 mg/kg DON for 21 d. In this study, ADG declined 7.4% per mg/kg and ADFI declined 8.0% per mg/kg increase in dietary DON when expressed relative to performance at 0.2 mg/kg. These proportional reductions exceed previously reported summary estimates of approximately 4.6% for ADG and 6.7% for ADFI per mg/kg DON in nursery pigs (Etienne and Wache 2008). This comparison suggests that growth performance responses to DON may be more pronounced in young, lightweight pigs used in controlled titration studies, while meta-analytical estimates represent composite responses across pigs of varying body weights and physiological stages, potentially diluting age- or size-dependent sensitivity.

A meta-analysis by Andretta et al. (2012) reported that dietary DON exposure is strongly correlated to reduced ADFI and ADG, while increasing feed efficiency (*R*^2^ = 0.87, 0.57, and 0.43, respectively) in studies combining nursery and grower pigs. Data from the present study are consistent with these observations, where DON concentration accounted for 58%, 48%, and 39% of the variation in overall ADFI, ADG, and BW, respectively, indicating that reduced feed intake was the primary driver of impaired growth. Only 64% of the studies included in Andretta et al. (2012) were conducted in nursery pigs; the remaining 36% performed in grow-finish pigs, which may partially inflate the reported feed efficiency relationship. This may reflect differences in effective DON exposure relative to body weight, as younger pigs receive a greater dose per unit of body weight compared to heavier pigs when consuming diets with similar DON concentrations, potentially resulting in a more pronounced response. Additionally, data compiled from eight studies showed an 8.45% decrease in weight gain per mg/kg DON (*R*^2^ = 0.66) (Dersjant-Li et al. 2003).

Across the 24-d period, cumulative BW remained linearly suppressed, demonstrating that early intake depression exerts persistent effects on overall performance. These findings align with the well-established anorectic effect of DON, in which reduced voluntary feed intake has been consistently identified as the principal mechanism underlying growth suppression in pigs (Dänicke et al. 2004; Frobose et al. 2015a; Wellington et al. 2020). While DON can impair intestinal integrity and immune function (Ghareeb et al. 2015), these gastrointestinal disruptions did not result in consistent changes in feed efficiency in the present study (*R*^2^ = 0.01), indicating that reduced voluntary feed intake, rather than impaired nutrient utilization, was the primary factor limiting growth performance within this exposure range.

In conclusion, increasing dietary DON concentration from 0.2 to 5.1 mg/kg resulted in clear, linear, and dose-dependent suppression of nursery pig growth performance. Reductions in ADG and ADFI were linear across the evaluated range, with no evidence of a biologically meaningful breakpoint within the DON dose range tested. However, one could speculate that at higher dietary DON concentrations above 5.1 mg/kg, a biological breakpoint could be plausible. When expressed relative to the lowest dietary concentration (0.2 mg/kg), ADG and ADFI declined by 7.4% and 8.0% per mg/kg DON, respectively. Ultimately, translating to slower time to market, increased days on feed, and higher production costs per pig. Growth suppression was driven primarily by progressive reductions in voluntary feed intake, as feed efficiency was not associated with DON concentration. Collectively, the linear exposure response relationship defined in this study provides a quantitative framework for interpreting DON associated performance losses and establishes a foundation for subsequent mechanistic investigations aimed at determining the biological pathways driving DON induced anorexia and growth suppression. These findings provide context for interpreting DON contamination risk relative to FDA guidance, demonstrating that growth suppression can occur at or below recommended limits and highlighting the importance of minimizing dietary exposure in nursery pig diets. The hypothesis that voluntary feed intake and growth performance would be reduced in a quadratic or segmented manner was rejected, as responses followed a linear pattern across the evaluated DON concentrations.

## List of abbreviations

ADFI: average daily feed intake
ADG: average daily gain
AIC: Akaike’s information criterion
BIC: Bayesian information criterion
BW: body weight
DON: deoxynivalenol
G:F: gain:feed
LC: liquid chromatography
LC-MS/MS: liquid chromatography-tandem mass spectrometry
lm: linear model
MS: mass spectrometry
MS/MS: tandem mass spectrometry
SID: standardized ileal digestible
